# Prognostic and predictive factors in patients with metastatic or recurrent cervical cancer treated with platinum-based chemotherapy.

**DOI:** 10.1186/s12885-017-3435-x

**Published:** 2017-06-28

**Authors:** Sofia Karageorgopoulou, Ioannis D. Kostakis, Maria Gazouli, Sonia Markaki, Marios Papadimitriou, Evangelos Bournakis, Meletios-Athanassios Dimopoulos, Christos A Papadimitriou

**Affiliations:** 1 Oncology Unit, 2nd Department of Surgery, Aretaieio Hospital, Medical School, National and Kapodistrian University of Athens, V. Sophias 76, 11528 Athens, Greece; 2 2nd Dept of Propedeutic Surgery, “Laiko” General Hospital, Medical School, National and Kapodistrian University of Athens, Athens, Greece; 3 Department of Biology, Medical School, National and Kapodistrian University of Athens, Athens, Greece; 4 Department of Pathology, Alexandra Hospital, Athens, Greece; 5 Department of Clinical Therapeutics, Alexandra Hospital, Medical School, National and Kapodistrian University of Athens, Athens, Greece

## Abstract

**Background:**

Recognizing resistance or susceptibility to the current standard cisplatin and paclitaxel treatment could improve therapeutic outcomes of metastatic or recurrent cervical cancer.

**Methods:**

Forty-five tissue samples from patients participating in a phase II trial of cisplatin and ifosfamide, with or without paclitaxel were collected for retrograde analysis. Immunohistochemistry and genotyping was performed to test ERCC1, III β-tubulin, COX-2, CD4, CD8 and *ERCC1* (C8092A and N118 N) and *MDR1* (C3435T and G2677 T) gene polymorphisms, as possible predictive and prognostic markers. Results were statistically analyzed and correlated with patient characteristics and outcomes.

**Results:**

Patients with higher levels of ERCC1 expression had shorter PFS and OS than patients with low ERCC1 expression (mPFS:5.1 vs 10.2 months, *p* = 0.027; mOS:10.5 vs. 21.4 months, *p* = 0.006). Patients with TT in the site of *ERCC1* N118 N and GT in the site of *MDR1* G2677 T polymorphisms had significantly longer PFS (*p* = 0.006 and *p* = 0.027 respectively). ERCC1 expression and the *ERCC1* N118 N polymorphism remained independent predictors of PFS. Interestingly, high III beta tubulin expression was associated with chemotherapy resistance and fewer responses [5/20 (25%)] compared to lower III β-tubulin expression [15/23 (65.2%)] (*p* = 0.008). Finally, ΙΙΙ β-tubulin levels and chemotherapy regimen were independent predictors of response to treatment.

**Conclusions:**

ERCC1 expression proved to be a significant prognostic factor for survival in our metastatic or recurrent cervical cancer population treated with cisplatin based chemotherapy. *ERCC1* N118 N and *MDR1* G2677 T polymorphism also proved of prognostic significance for disease progression, while overexpression of III β-tubulin was positively correlated with chemotherapy resistance.

## Background

Cancer of the uterine cervix represents the fourth most common malignancy among females and accounts for 7.5% of all cancer deaths in women worldwide. Due to lack of systemic screening programs, developing countries share the 85% of the global burden, with cervical cancer accounting for 12% of all cancers among women in these countries [1]. Patients with metastatic or recurrent cervical cancer are treated mainly with palliative chemotherapy. In this setting, cisplatin may be combined with either paclitaxel, topotecan, gemcitabine or vinorelbine, since no significant differences in OS (overall survival) have been observed between these regimens, although survival trends and toxicity profiles seem to favor the cisplatin and paclitaxel combination [2, 3]. Lately, significant therapeutic progress has been documented by adding the antiangiogenic agent bevacizumab to the standard cisplatin-paclitaxel or topotecan-paclitaxel regiments, that extended median OS from 13.3 to 17 months, as shown in the GOG 204 trial [4]. However, one should keep in mind that this gain comes with a significant incremental cost-effectiveness ratio (ICER) that is over $120,000/quality adjusted life year (QALY), almost double than the willingness-to-pay (WT) of $50,000–$62,500/QALY in the US, according to recent cost-effectiveness studies [5].

Resistance to chemotherapy is widely recognized as one of the major factors that limit therapeutic efficacy and influence patient outcomes. Cisplatin and carboplatin are alkylating compounds that exert their cytotoxic action by binding to DNA and forming strong inter- and intra-structural cross links, thus inhibiting DNA replication [6]. Excision repair cross-complement 1 (*ERCC1*) is a 15-kb human nucleotide excision repair gene with already documented importance in developing resistance to platinum compounds in NSCLC (non small cell lung cancer), ovarian, colorectal and cervical cancer [7–11]. Most of the ERCC1 genes studied are polymorphic. These SNPs may or not alter the protein function. Even if they do not result in an amino acid change they may cause mRNA instability and increase the risk of environmentally induced cancer [12].

Class III tubulin is a common target for taxane chemotherapy and its overexpression has been associated with resistance in patients with NSCLC, breast cancer and gastric cancer treated with tubulin binding agents [13]. The Multiple Drug Resistance 1 (*MDR-1*) gene is a highly polymorphic gene that codes for the membrane transporter P-glycoprotein and its variations have been associated with influenced protein function, altered kinetics of anticancer drugs and respective patient outcomes [14–16]. Moreover, it has been described that cyclooxygenase 2 (COX-2) plays a role in carcinogenesis of cervical, ovarian and endometrial neoplasms by inhibiting surveillance by the immune system, neo-angiogenesis and apoptosis [17–19]. Efficient immune response requires activation of CD4 and CD8 T lymphocytes and activation of tumor-infiltrating cytotoxic T lymphocytes is correlated with improved survival in cervical, endometrial, ovarian, pancreatic and colorectal cancers [20–24].

The above markers were chosen based on their previous correlation with survival in locally advanced cervical cancer (LACC) and other cancer subtypes, as well as on previous references associating them with platinum or taxane resistance. We did not attempt to make a gene signature. The aim of this study was to confirm or not the prognostic and or predictive value of these specific markers in the metastatic and or recurrent cervical cancer setting. Specifically we tested whether ERCC1 expression and two frequently described SNPs (single nucleotide polymorphisms) *ERCC1* (C8092A and N118 N) could predict response and clinical outcomes in metastatic or recurrent cervical cancer patients treated with cisplatin-based chemotherapy. We also evaluated if there are any associations between the two common polymorphisms in *MDR1* gene (C3435T and G2677 T), as well as class III β-tubulin with survival and chemotherapy resistance in the same population. Finally, we looked for possible correlations between tumor microenvironment expression of COX-2, and percentage of CD4 and CD8 tumor infiltrating lymphocytes (TILs) with patient characteristics and clinical outcomes.

## Methods

### Patient selection

Tissue samples from patients that participated in a randomized multicenter phase II trial of cisplatin and ifosfamide with or without paclitaxel were provided for retrograde analysis. This trial randomly allocated one hundred and fifty-three patients to receive either ifosfamide 1.5 g/m^2^, daily, on days 1–3 and cisplatin 70 mg/m^2^ on day 2 (IP regimen) or the same combination with the addition of paclitaxel 175 mg/m^2^, on day 1 (ITP regimen), every 4 weeks [25]. Retrograde immunohistochemical analysis and genotyping was performed to eventually 45 available tissue samples, as well as correlation with patient characteristics and outcomes. World Health Organization criteria for response were used [26]. Eligible patients had primary metastatic or recurrent carcinoma of the uterine cervix, not amenable to surgery and/or radiation therapy and had not been treated with prior chemotherapy except for cisplatin chemo-radiation.

### Immunohistochemistry

Tissue samples were removed and embedded in 10% neutral–buffered formalin. Sections were then dehydrated in graded series of ethanol concentrations of 50%, 60%, 70%, 80%, 90% and 100%, respectively. The tissue intubation time in each ethanol solution was 90 min. Subsequently, the tissue was embedded in 2 xylene and 3 alcohol buffers for 90 min each. The whole procedure lasted 18 h.

Tissue fixation followed in paraffin blocks and sections of 4 μm were cut and placed on specific ionized slides (SuperFrost™ Plus) in order to avoid their autoagglutination. Immunohistochemistry was performed on an automated immunohistochemistry system (Bond-Max, Leica). The required dewax and antigen retrieval procedures were both automated and performed by the use of Bond Dewax Solution and Bond Epitope Retrieval Solution 1 and 2 (Leica Biosystems), respectively. For antibody labeling the Bond Polymer Refine Red Detection Kit (Leica Biosystems) was used. Staining was achieved through the Fast Red Chromogen System (BioLegend), and counterstaining through a 0.02% haematoxylin solution. Finally, tissue dehydration in graded alcohol and xylene was done and microscopic examination was performed. The following monoclonal antibodies were used: For ΕRCC1, IgG2b, clone 8F1(1:70) and for COX-2, IgG1, clone 4H12, (1:30) (both Diagnostic BioSystems Inc., Pleasanton, CA, USA). For III β-tubulin, IgG1, clone OTI5H2 (1:70) (Acris Antibodies Inc., San Diego, CA, USA). For CD4, IgG1 antibody, clone 4B12, (1:80) and for CD8, IgG1, clone 1A5, (1:20) (both Novocastra Inc.).

### Staining evaluation

Two independent pathologists who were blinded for patient’s identity, characteristics and outcomes performed the immunochemistry assessment. Positive reaction was expressed based on the percentage of tumor cells with membrane staining (0: 0%; 1: 0–10%; 2: 10–50%; 3: >50% of stained tumor cells). We considered as positive the samples with over 50% of tumors cells stained. A third pathologist reviewed discordant cases.

### Genotyping

Genotyping of *ERCC1* C8092A and N118 N were determined by using the polymerase chain reaction-restriction fragment length polymorphism (PCR-RFLP) assay as previously described [27, 28]. The primers used were: For the C8092A, 8092F: 5′-ACCCCACTCTAGATTTACCCAGGAA-3′ and 8092R: 5′-AAGAAGCAGAGTCAGGAAAGC-3′. The PCR products were digested with the restriction enzyme MboII. For the N118 N polymorphism 118F: 5′-AGGACCACAGGACACGCAGA-3′ and 118R: 5′-CATAGAACAGTCCAGAAC AC-3′, respectively. The PCR products were digested with restriction enzyme BsrdI to determine the genotypes.

Genotyping of *MDR1* C3435T (exon 26) and G2677 T (exon 21) were also determined by using the PCR-RFLP assay as previously described [29, 30]. Specifically, PCR amplifications were carried out in a total volume of 50 μl containing: 100 ng of genomic DNA, 1 U of Taq Polymerase (MBI Fermentas), 1 μM of each primer (for C3435T, F: 5′-TTG ATG GCA AAG AAA TAA AGC-3′ and R: 5′-CTT ACA TTA GGC AGT GAC TCG-3′; for G2677 T, F: 5′-TTT GCA GGC TAT AGG TTC CAG-3′, and R(T): 5′-TTT AGT TTG ACT CAC CTT CCC G-3′), 1XPCR buffer, 1 mM MgCl_2_, and 0.04 mM dNTPs. The PCR products were digested by restriction endonucleases MboI (for C3435T) and BanI (for G2677 T).

### Statistical analysis

Chi-square test and Fisher’s exact test were used for comparisons between groups with categorical variables. Multivariate analysis for predictors of categorical dichotomous outcomes was performed with logistic regression. Overall and progression-free survivals (PFS) were estimated with the Kaplan-Meier method, which was also used for comparisons of survivals among different groups. Multivariate survival analysis was performed with Cox regression with the forward conditional method. All tests were two-tailed. The results were considered statistically significant if *p* < 0.05.

## Results

### Demographics

Main patients’ characteristics are summarized in Table 1. The median patient age was 58 years (range 32–76). Squamous cell carcinoma accounted for 72.1% (*n* = 31), followed by adenocarcinoma (*n* = 6, 14%), and mixed histological types (*n* = 6, 14%). Of the total 43 patients, 22 received the ITP regimen and 21 the IP regimen. 42 out of the 43 patients (97.7%) showed disease progression during the surveillance period and thirty-seven out of the 43 patients (86%) died. The median PFS of the patients in our cohort was 6 months (range: 0.2–57.3 months) and the median OS was 11.6 months (range: 0.2–81 months). Data on immunohistochemistry expression of the tested proteins and selected single nucleotide polymorphisms of this metastatic or recurrent cervical cancer cohort is summarized in Tables 2 and 3 respectively.

**Table 1:** Selected patient characteristics

Characteristic	No of patients (%)		*p*-value
	ITP	IP	
Total patients	22	21	
Age (years)			
Median	58	58	0.646
Range	32–78	35–75	
Histology			
Squamous	13 (59.1)	18 (85.7)	0.129
Adenocarcinoma	4 (18.2)	2 (9.5)	
Mixed	5 (22.7)	1 (4.8)	
Overall response			
CR	5 (22.7)	1 (4.8)	0.038
PR	10 (45.5)	4 (19)	
SD	2 (9.1)	5 (23.8)	
PD	5 (22.7)	11 (52.4)	

*ITP* Ifosfamide Paclitaxel Cisplatin, *IP* Ifosfamide Cisplatin, *CR* Complete Response, *PR* Partial Response, *SD* Stable Disease, *PD* Progressive Disease

**Table 2:** Immunohistochemistry patient data

Protein Expression	No of patients (%)		*p*-value
	ITP	IP	
ERCC 1			
High	11 (50)	9 (42.9)	0.906
Moderate	5 (22.7)	7 (33.3)	
Low	2 (9.1)	2 (9.5)	
None	4 (18.2)	3 (14.3)	
COX 2			
High	7 (31.8)	5 (23.8)	0.342
Moderate	5 (22.7)	7 (33.3)	
Low	4 (18.2)	7 (33.3)	
None	6 (27.3)	2 (9.5)	
III beta tubulin			
High	11 (50)	9 (42.9)	0.425
Moderate	8 (36.4)	5 (23.8)	
Low	3 (13.6)	6 (28.6)	
None	0 (0)	1 (4.8)	
CD4			
High	0 (0)	0 (0)	0.768
Moderate	3 (13.6)	4 (19)	
Low	6 (27.3)	7 (33.3)	
None	13 (59.1)	10 (47.6)	
CD8			
High	1(4.5)	0 (0)	0.226
Moderate	3 (13.6)	3 (14.3)	
Low	3 (13.6)	8 (38.1)	
None	15 (68.2)	10 (47.6)	

*ITP* Ifosfamide Paclitaxel Cisplatin, *IP* Ifosfamide Cisplatin

**Table 3:** Selected single nucleotide polymorphisms patient data

SNPs	No of patients (%)		*p*-value
	ITP	IP	
*MDR1* C3435T			
Polymorphisms			
CT	13 (59.1)	14 (66.6)	1
CC	4 (18.2)	3 (14.3)	
TT	5 (22.7)	4 (19)	
*MDR1* G2677 T			
Polymorphisms			
GT	5 (22.7)	5 (23.8)	0.904
GG	15 (68.2)	13 (61.9)	
TT	2 (9.1)	3 (14.3)	
*ERCC1* C8092A			
Polymorphisms			
CA	9 (40.9)	8 (38.1)	1
CC	11 (50)	10 (47.6)	
AA	2 (9.1))	3 (14.3)	
*ERCC1* N118 N			
Polymorphisms			
CT	8 (36.4)	12 (57.1)	0.371
CC	3 (13.6)	3(14.3)	
TT	11 (50)	6 (28.6)	

*ITP* Ifosfamide Paclitaxel Cisplatin, *IP* Ifosfamide Cisplatin

### Protein expression association with patient characteristics

Histological type of cervical cancer seemed to be associated with COX2 and CD8 protein expression. COX2 was expressed in the great majority of squamous carcinomas (90.3%) and in 66.7% and 50% of adenosquamous and adenocarcinomas respectively (*p* = 0.034). Although of marginal statistical significance (*p* = 0.05), CD8 was also more abundantly expressed in squamous carcinomas (51.6%) than in adenocarcinomas (33.3%) and adenosquamous carcinomas (0%). No significant associations were found between age and the expression of ERCC1 (*p* = 0.706), COX2 (*p* = 0.731), tubulin B3 (*p* = 0.529), CD4 (*p* = 0.515) or CD8 (*p* = 0.281) TILs.

### Genotype distributions and their association with patient characteristics and protein expression

Similarly, no significant associations were found between age or histological type the presence of the following polymorphisms: *MDR1* C3435T (*p* = 0.253), *MDR1* G2677 T (*p* = 0.609), *ERCC1* C8092A (*p* = 1), *ERCC1* N118 N (*p* = 0.684). On the contrary, the polymorphism *ERCC1* N118 N seemed to influence the production of ERCC1, since all the tumors with CT genotype were stained positive for ERCC1 protein [20/20 (100%)], whereas this was not the case for the other two tested alternatives [CC: 4/6 (66.6%), TT: 12/17 (70.6%)] (*p* = 0.013).

### Immunohistochemisrty associations with response and survival outcomes

As it has been previously published, patients on the ITP regimen demonstrated significantly higher response to chemotherapy and improved survival outcomes [25]. In our study, no significant correlations were observed between the response rates and the levels of ERCC1 expression (*p* = 0.13). Responses were influenced by the expression of some of the other examined proteins. Specifically, patients with high ΙΙΙ β-tubulin expression demonstrated decreased complete or partial responses [5/20 (25%)] compared to patients with lower or no expression of ΙΙΙ β-tubulin [15/23 (65.2%)] (*p* = 0.008). The type of chemotherapy regimen and the levels of ΙΙΙ β-tubulin remained independent predictors of response to the treatment after multivariate analysis using logistic regression. In particular, patients having received the ITP regimen had more objective responses than patients having received the IP regimen [HR = 22.45 (95% CI: 2.486–202.725), *p* = 0.006,] and patients with high ΙΙΙ β-tubulin expression had less objective responses than patients with lower or no ΙΙΙ β-tubulin expression [HR = 0.52 (95% CI: 0.006–0.469) *p* = 0.008]. Five out of eleven patients (45.5%) in the ITP regimen and five out of twelve (541.7%) patients in the IP regimen had progressing disease (PD) when ΙΙΙ β-tubulin was overexpressed compared to 0% of patients progressing in either treatment arms when lower or absent III β-tubulin was expressed (*p* = 0.035 and *p* = 0.045 for the ITP and IP respectively).

**Fig. 1:**
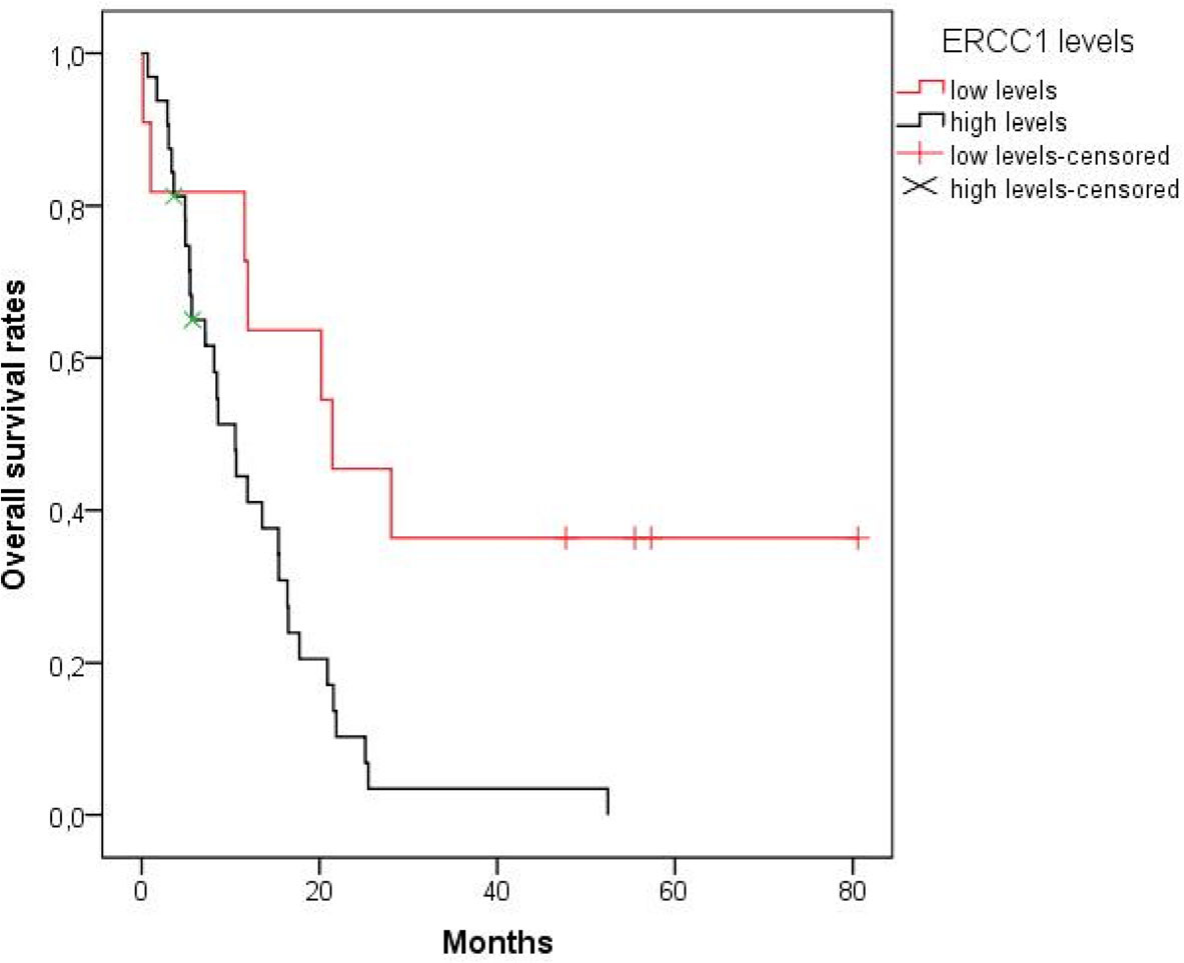
OS according to ERCC1 expression. Patients with moderate or high levels of ERCC1 had shorter overall survival [median OS: 10.5 months mean OS ± SE: 12.5 months ±1.9 (95% CI: 8.8–16.3),] than patients with low or no ERCC1 production [median OS: 21.4 months, mean OS ± SE: 37.9 months ±10 (95% CI: 18.3–57.5)] (*p* = 0.006). OS, Overall Survival

**Fig. 2:**
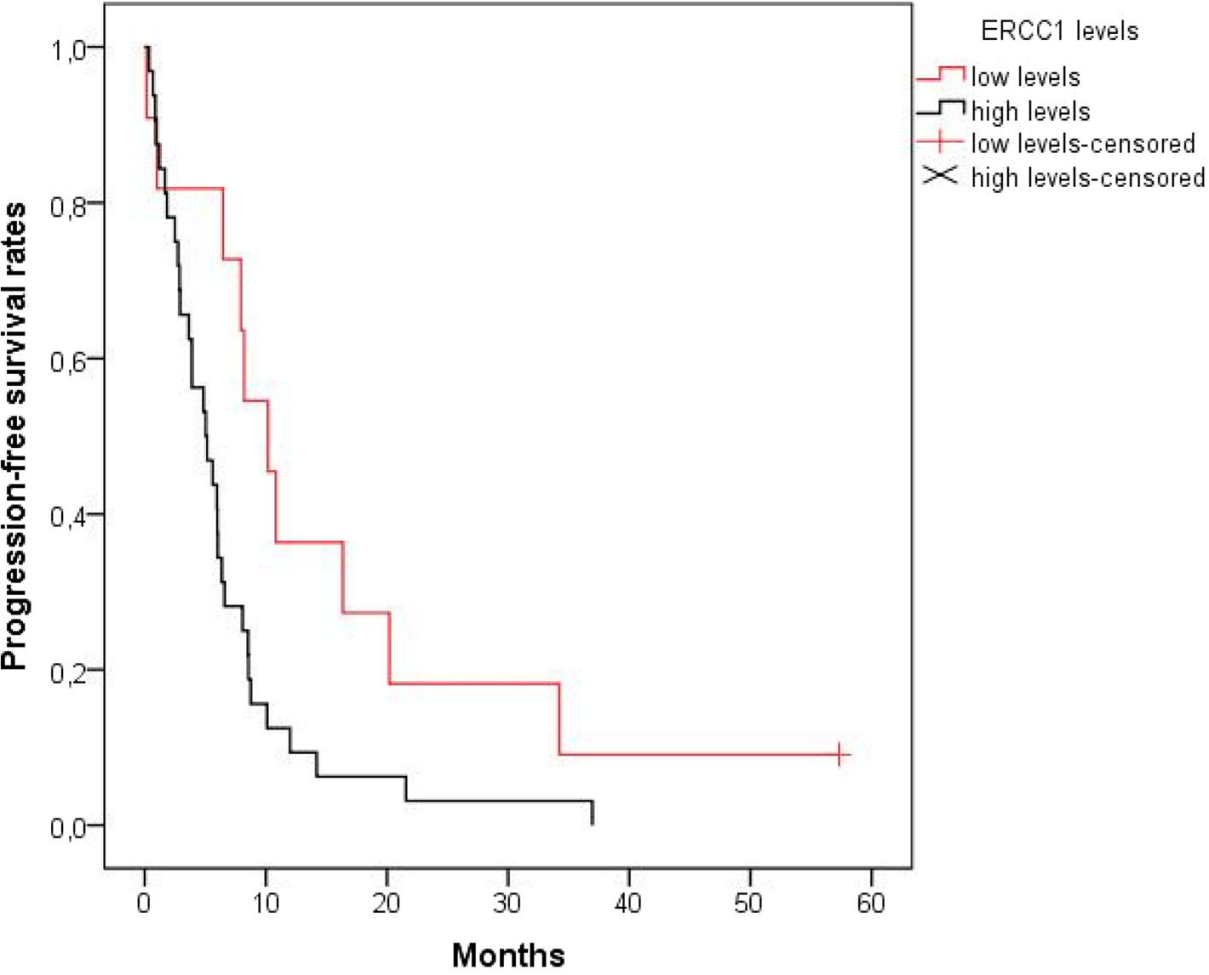
PFS according to ERCC1 expression. Patients with moderate or high levels of ERCC1 had shorter progression-free survival [median PFS: 5.1 months, mean PFS ± SE: 6.6 months ±1.3 (95% CI: 4.1–9)] than patients with low or no ERCC1 production [median PFS: 10.2 months, mean PFS ± SE: 15.7 months ±4.8 (95% CI: 6.3–25.2)] (*p* = 0.027). PFS, Progression Free Survival

PFS and OS according to the tested parameters are summarized in Tables 4, 5 and 6, for the ITP + IP, ITP and IP groups respectively. III β-tubulin expression did not significantly affect OS or PFS in either ITP or IP group. However, ERCC1 expression showed a strong negative correlation with PFS and OS in this metastatic cervical cancer cohort. Median OS for patients with high or moderate levels of ERCC1 was 10.5 months versus 21.4 months for patients with low or no ERCC1 production (*p* = 0.006) (Fig. 1). Median PFS was also significantly shorter in patients with ERCC1 overexpression (mPFS: 5.1 months vs 10.2 months respectively, *p* = 0.027) (Fig. 2). When we conducted multivariate survival analysis using Cox regression, only ERCC1 expression remained an independent predictor of both the OS [HR = 3.187 (95% CI: 1.346–7.546), *p* = 0.008,] and the PFS [HR = 2.473 (95% CI: 1.146–5.339), *p* = 0.021].

**Table 4:** OS and PFS (all patients)

	Median OS (months)				Median PFS (months)			
Protein Expression	Low	High		*P*-value	Low	High		*P*-value
ERCC1	21.4	10.5		0.006	10.2	5.1		0.027
COX2	17.7	10.5		0.051	6.5	5.2		0.463
Tubulin B3	11.6	11.9		0.704	6	6		0.347
CD4	11.9	11.9		0.446	5.6	8.8		0.253
CD8	13.5	8.6		0.041	6	3.9		0.766
SNPs								
*MDR1* C3435T	CC	TT	CT	*P*-value	CC	TT	CT	*P*-value
	20.2	16.5	10.5	0.19	7.9	6.6	6	0.654
*MDR1 G2677 T*	GG	TT	GT		GG	TT	GT	
	8.6	3.6	17.7	0.119	5.1	2.9	8.6	0.027
*ERCC1C8092A*	AA	CC	CA		AA	CC	CA	
	25.2	11.6	15.4	0.756	2.8	6	6	0.543
*ERCC1 N118 N*	CC	TT	CT		CC	TT	CT	
	8.2	21.4	8.5	0.063	5.2	8.8	3.9	0.006

**Table 5:** OS and PFS (ITP group)

	Median OS (months)				Median PFS (months)			
Protein Expression	Low	High		*P*-value	Low	High		*P*-value
ERCC1	21.4	10.5		0.049	8.2	6		0.558
COX2	17.7	10.5		0.363	7.9	6		0.895
Tubulin B3	11.9	16.4		0.529	8.2	7.9		0.74
CD4	11.9	25.5		0.397	6.6	10.1		0.113
CD8	11.9	5.4		0.476	7.9	1.2		0.707
SNPs								
MDR1 C3435T	CC	TT	CT	*P*-value	CC	TT	CT	*P*-value
	3.4	11.9	11.7	0.467	1.8	8.8	8.1	0.484
MDR1 G2677 T	GG	TT	GT		GG	TT	GT	
	11.9	2.9	21.9	0.647	6.5	2.9	8.6	0.494
ERCC1C8092A	AA	CC	CA		AA	CC	CA	
	25.2	11.9	11.9	0.664	10.2	7.9	6.5	0.702
ERCC1 N118 N	CC	TT	CT		CC	TT	CT	
	11.6	21.6	5.6	0.401	8.2	10.1	3.6	0.003

**Table 6:** OS and PFS (IP group)

	Median OS (months)				Median PFS (months)			
Protein Expression	Low	High		*P*-value	Low	High		*P*-value
ERCC1	20.2	8.6		0.114	20.2	3.9		0.045
COX2	15.4	8.6		0.106	5.1	2.9		0.362
Tubulin B3	5.4	10.6		0.349	4.9	3.9		0.419
CD4	8.6	3.6		0.946	4.9	2.8		0.81
CD8	8.6	15.4		0.919	4.9	3.9		0.924
SNPs								
MDR1 C3435T	CC	TT	CT	*P*-value	CC	TT	CT	*P*-value
	13.5	15.4	8.2	0.318	8.5	4.9	2.8	0.374
MDR1 G2677 T	GG	TT	GT		GG	TT	GT	
	8.2	3.6	15.4	0.104	3.9	2.8	12	0.041
ERCC1C8092A	AA	CC	CA		AA	CC	CA	
	3.6	8.6	15.4	0.14	0.9	3.9	5.1	0.008
ERCC1 N118 N	CC	TT	CT		CC	TT	CT	
	8.2	10.6	8.5	0.394	5.1	6.4	3.9	0.195

Moreover, patients without any CD8 TILs expression in their tumors had a more favorable OS profile than patients with tumors expressing CD8 at any grade (mOS: 13.5 vs 8.6 months respectively, *p* = 0.041) (Table 4). Patients with higher levels of COX2 expression tended to had shorter OS than patients with low or no COX2 production (mOS: 10.5 vs 17.7 months respectively, *p* = 0.051).

### Genotypic polymorphisms and their associations with response and survival outcomes and relevant protein expression

No significant correlations were observed between the response rates and the tested polymorphisms: *MDR1* C3435T (*p* = 0.867), *MDR1* G2677 T (*p* = 0.191), *ERCC1* C8092A (*p* = 0.454), *ERCC1* N118 N (*p* = 0.479).

Interestingly, the presence of E*RCC1* N118 N polymorphism seemed to translate in ERCC1 protein expression, since all the tumors with the CT genotype were stained positive for ERCC1 protein [20/20 (100%)]. This was not the case for the other two genotypes [CC: 4/6 (66.6%), TT: 12/17 (70.6%)] (*p* = 0.013) or *ERCC1* C8092A polymorphisms that did not show to affect ERCC1 protein levels (*p* = 0.358).

**Fig. 3:**
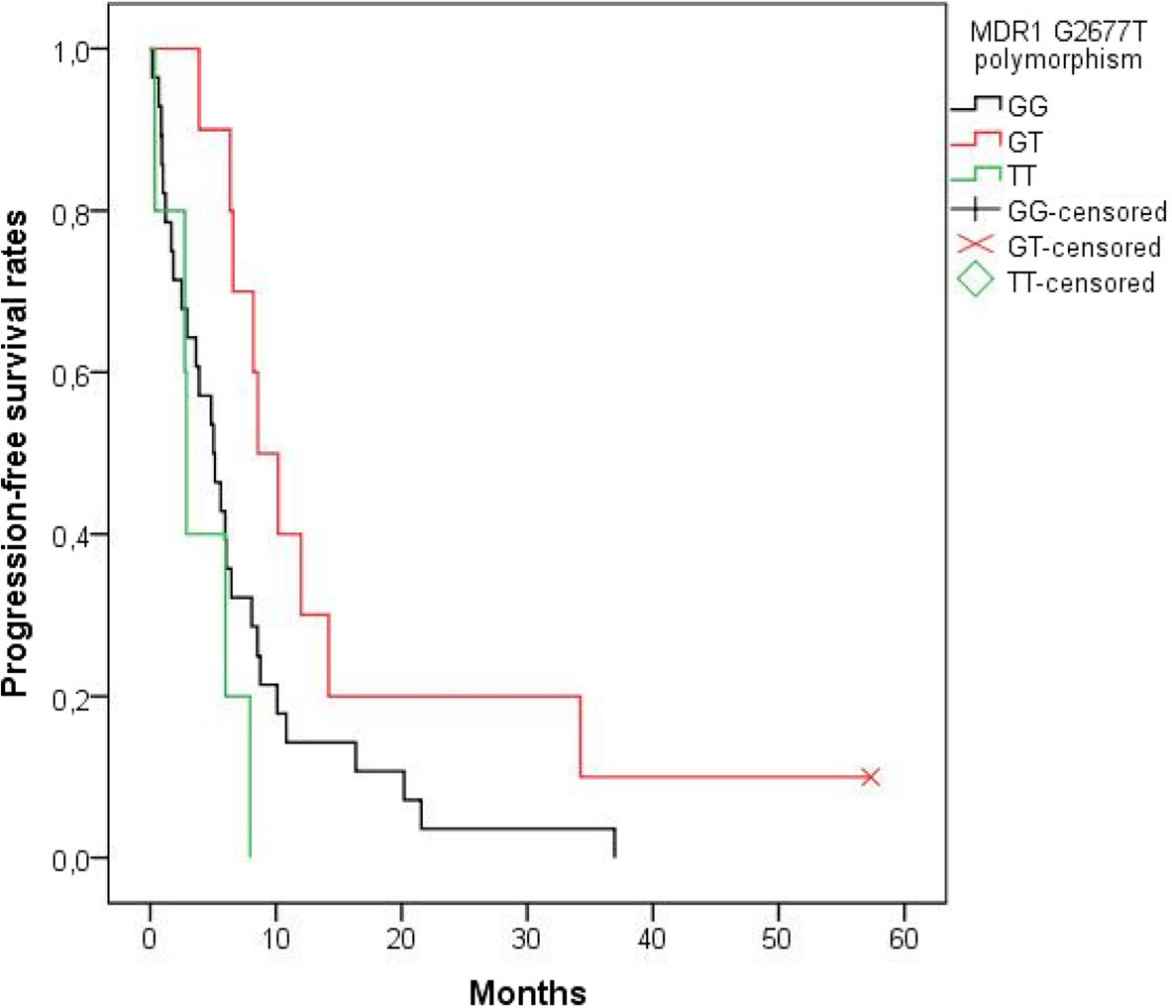
PFS according to *MDR1* G2677 T polymorphism. Patients with GT in the site of the *MDR1* G2677 T polymorphism lived longer without disease progression [median PFS: 8.6 months, mean PFS ± SE: 16.2 months ±5 (95% CI: 6.3–26)] than patients with GG at the same site [median PFS: 5.1 months, mean PFS ± SE: 7.2 ± 1.5 (95% CI: 4.2–10.20)], who in turn had longer progression-free survival than patients with TT at the same site [median PFS: 2.9 months, mean PFS ± SE: 4 months ±1.3 (95% CI: 1.4–6.6)] (*p* = 0.027). PFS, Progression Free Survival

**Fig. 4:**
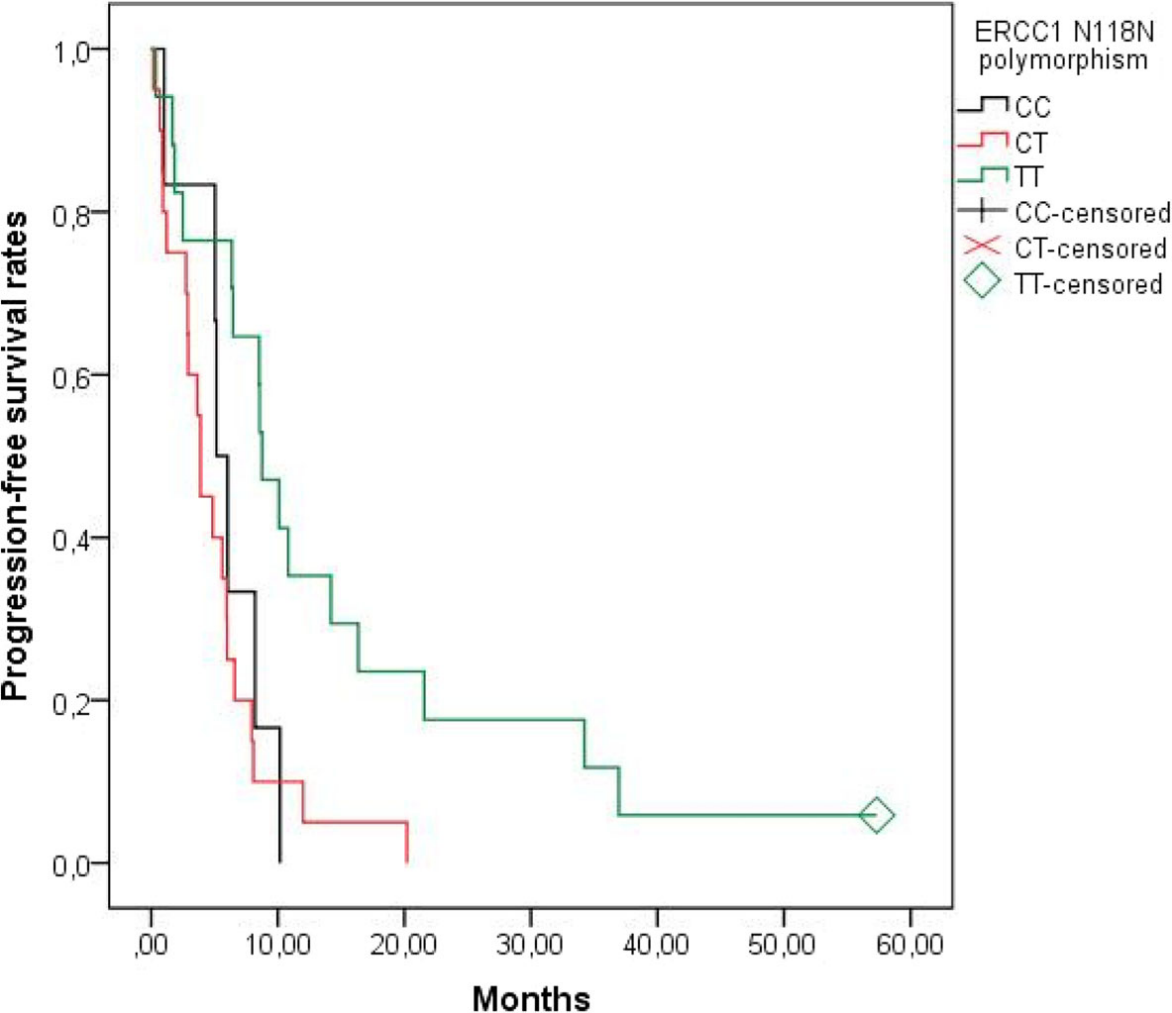
PFS according to *ERCC1* N118 N polymorphism. Patients with TT in the site of the *ERCC1* N118 N polymorphism lived longer without disease progression [median PFS: 8.8 months, mean PFS ± SE: 14.5 months ±3.6 (95% CI: 7.5–21.5)] than patients with CC at the same site [median PFS: 5.2 months, mean PFS ± SE: 5.9 ± 1.3 (95% CI: 3.4–8.4)] and patients with CT at the same site [median PFS: 3.9 months, mean PFS ± SE: 5.1 months ±1 (95% CI: 3–7.1)] (*p* = 0.006). PFS, Progression Free Survival

On the contrary, *MDR1* G2677 T and *ERCC1* N118 N genetic polymorphisms examined in the study appeared to influence the median PFS of patients with metastatic or recurrent cervical cancer. Patients with GT in the site of the *MDR1* G2677 T polymorphism demonstrated longer intervals without disease progression (mPFS: 8.6 months) than patients with GG at the same site (mPFS: 5.1 months), who in turn had longer PFS than patients with TT at the same site (mPFS: 2.9 months, *p* = 0.027) (Fig. 3). In addition, patients with TT in the site of the *ERCC1* N118 N polymorphism lived longer without disease progression (mPFS: 8.8 months) than patients with CC at the same site (mPFS: 5.2 months) and patients with CT at the same site (mPFS: 3.9 months, *p* = 0.006) (Fig. 4). Finally, PFS was not affected significantly by the genetic polymorphisms *MDR1* C3435T (*p* = 0.654) or *ERCC1* C8092A (*p* = 0.543). Moreover, the *ERCC1* N118 N polymorphism still was a strong predictor of disease progression (*p* = 0.007) after multivariate analysis.

## Discussion

Metastatic or recurrent cancer of the uterine cervix remains a major cause of death for women. These patients are mainly treated with palliative chemotherapy and their prognosis remains extremely poor. Therefore, recognizing resistance or susceptibility to the current standard cisplatin and paclitaxel based treatment may improve patient outcomes and direct selected patients to other new possible options such as immunotherapy or targeted agents. Back in 2000, Britten et al. described a statistically significant (*p* < 0.011) association between high ERCC1 mRNA levels and cisplatin resistance in human cervical cancer cell lines. Thereafter, several studies tested ERCC1 as a possible marker of resistance in cervical cancer [31]. High ERCC1 expression was a poor prognostic factor and was correlated with poor disease-free survival (DFS) (*p* = 0.021) and OS (*p* = 0.005) in 88 locally advanced cervical cancer (LACC) patients who received cisplatin monotherapy as reported by Zwenger et al. [32]. Similarly, class III-β tubulin did not demonstrate a significant association with response, nor prognosis in a series of 98 LACC patients subjected to concurrent chemoradiotherapy [33]. Accordingly, in a larger Canadian study including 264 LACC patients undergoing curative chemoradiation, ERCC1 expression was positively correlated with PFS (HR 2.33 [1.05–5.18], *P* = .038) and OS (HR 3.13 [1.27–7.71], *P* = .013), but was not an independent prognostic factor [34]. Interestingly enough, the same group showed that ERCC1 expression was significantly correlated with both OS (*p* = 0.002) and DFS (*p* = 0.010) among 186 patients undergoing radical radiotherapy alone [35]. Worse DFS was also documented among 25 patients with FIGO IB – IIB cervical cancer who underwent either concurrent chemoradiotherapy with cisplatin or cisplatin-based chemotherapy and demonstrated high ERCC1 protein expression (*P* = 0.002) [36]. Similar results have been reported also in the neoadjuvant setting among 43 stage IIB patients receiving etoposide and cisplatin. Park et al. showed that ERCC1 remained an independent negative predictive factor for response (*p* = 0.021) to cisplatin containing treatment [37]. Contradictory results have been also published by Muallem et al. in 112 LACC patients treated with cisplatin-based chemo-radiotherapy showing that high levels of ERCC1 expression correlated with favorable outcomes of patients [38].

To our knowledge, there has not been so far a description of the common *ERCC1* and *MDR1* gene SNPs associated with chemotherapy resistance and survival in recurrent or metastatic cervical cancer, nor its correlation with ERCC1 protein expression. Moreover, in the present study the chemotherapy backbone was cisplatin but also half of the patients were treated with paclitaxel, giving us the opportunity to explore resistance and outcome patterns to taxane chemotherapy. Indeed, the addition of paclitaxel (ITP regimen) did improve patient outcomes as described previously [25], and high III β-tubulin expression was associated with chemotherapy resistance, as it was linked with lower responses [5/20 (25%)] compared to lower expression of ΙΙΙ β-tubulin [15/23 (65.2%)] (*p* = 0.008). Although we recognize that the number of patients in our cohort are rather small, the multivariate analysis performed did show that the type of treatment, that is the addition of paclitaxel in ITP regimen (Ifosphamide, Paclitaxel, cisplatin) to IP ((Ifosphamide, cisplatin) did not confound the results, since high expression of III β-tubulin remained an independent predictor of response to treatment. If our results are confirmed in larger cohorts, testing III β-tubulin expression could provide a predictive tool for response to treatment and possibly guide those patients to enroll in clinical trials testing alternative treatment options

Surprisingly, ERCC1 protein expression and the examined *ERCC1* polymorphisms could not predict resistance to cisplatin based chemotherapy in this small metastatic or recurrent cervical cancer cohort. This may in part be due to the 8F1 antibody used for staining ERCC1. Recent data from the NSCLC setting suggest that this antibody cannot differentiate between the 4 isoforms of ERCC1 and more specifically the isoform 202 that is related to platinum sensitivity [39]. Another explanation would be that the results were underpowered due to the small number of patients in the study.

However, similarly to the studies in LACC, ERCC1 expression proved to be a significant prognostic factor for survival in our studied population. Patients with higher levels of ERCC1 had statistically shorter PFS and OS than patients with low ERCC1 expression (mPFS:5.1 vs 10.2 months, *p* = 0.027; mOS: 10.5 vs. 21.4 months, *p* = 0.006). In addition, the genetic polymorphisms *ERCC1* N118 N and *MDR1* G2677 T appear also to influence the PFS. Patients with TT in the site of the *ERCC1* N118 N polymorphism lived longer without disease progression than patients with CC or CT at the same site [(median PFS: 8.8, 5.2 and 3.9 months respectively, *p* = 0.006). Furthermore, patients with GT in the site of the *MDR1* G2677 T polymorphism lived longer without disease progression than patients with GG and patients with TT at the same site (median PFS: 8.6 months, 5.1 and 2.9 months respectively, *p* = 0.027). ERCC1 expression and the *ERCC1* N118 N polymorphism remained independent predictors of the PFS after the performed multivariate survival analysis, thus rendering them significant prognostic factors in this metastatic or recurrent cervical cancer population.

Finally, the absence of CD8 expression was also correlated with improved survival in our metastatic cervical cancer cohort. Although this merits further investigation, it is in concordance with the observation that it is the high CD4/CD8 ratio of tumor-infiltrating lymphocytes (TILs) and thus a low CD8 count, that is linked to improved survival of patients with cervical cancer [24]. Furthermore, in the later study, better clinical outcomes were shown when a high percentage of CD4 TILs combined with a low percentage of FOX 3 CD4 regulatory T cells was present [24].

## Conclusions

In conclusion, our data should be interpreted with caution given the small numbers of the cohort. However, these are in major concordance with previous data underlying the prognostic role of ERCC1 expression and its polymorphisms in the outcome of patients treated with platinum cytotoxic damaging agents. Furthermore, the efficacy of the already included paclitaxel in the standard treatment of metastatic cervical cancer may possibly be influenced by III β-tubulin expression and the described *MDR1* polymorphisms. In the new era of targeted therapies, the above information could be used to recognize specific subgroups of patients that would derive the major benefit from chemotherapy and those with poor prognosis that should be directed to clinical trials with novel promising agents.

## Abbreviations

COX-2: Cyclooxygenase 2
CR: Complete Response
ERCC1: Excision repair cross-complement 1
HR: Hazard ratio
IP: Ifosfamide Cisplatin
ITP: Ifosfamide Paclitaxel Cisplatin
LACC: Locally advanced cervical cancer
MDR-1: Multiple Drug Resistance
mOS: median overall survival
mPFS: median progression free survival
NSCLC: Non-small cell lung cancer
OS: Overall survival
PCR: Polymerase chain reaction
PD: Progressive Disease
PFS: Progression-free survival
PR: Partial Response
RFLP: Restriction fragment length polymorphism
SD: Stable Disease
SNPs: Single nucleotide polymorphisms

## References

[1] Ferlay J, Soerjomataram I, Dikshit R, Eser S, Mathers C, Rebelo M (2015). Cancer incidence and mortality worldwide: sources, methods and major patterns in GLOBOCAN 2012.

[2] Monk BJ, Sill MW, McMeekin DS (2009). Phase III trial of four cisplatin-containing doublet combinations in stage IVB, recurrent, or persistent cervical carcinoma: a gynecologic Oncology group study. J Clin Oncol.

[3] Long HJ, Bundy BN, Grendys EC (2005). Randomized phase III trial of cisplatin with or without topotecan in carcinoma of the uterine cervix: a gynecologic Oncology group study. J Clin Oncol.

[4] Tewari KS, Sill MW, Long HJ (2014). Improved survival with bevacizumab in advanced cervical cancer. N Engl J Med.

[5] Klag N, Walter AC, Sheely KM, Manahan KJ, Geisler JP (2016). Is the routine use of bevacizumab in the treatment of women with advanced or recurrent cancer of the cervix sustainable?. ClinicoEconomics and Outcomes Research: CEOR.

[6] Rice JA, Crothers DM, Pinto AL (1988). The major adduct of the antitumor drug cis-diamminedichloroplatinum (II) with DNA bends the duplex by approximate equal to 40 degrees toward themajor groove. ProcNatlAcad Sci U S A.

[7] Mountzios G, Dimopoulos M-A, Papadimitriou C (2008). Excision repair cross-complementation group 1 enzyme as a molecular determinant of responsiveness to platinum-based chemotherapy for non small-cell lung cancer. Biomark Insights.

[8] Smith S, Su D, Rigault de la Longrais IA, Schwartz P, Puopolo M, Rutherford TJ (2007). ERCC1 genotype and phenotype in epithelial ovarian cancer identify patients likely to benefit from paclitaxel treatment in addition to platinumbased therapy. J Clin Oncol.

[9] Britten RA, Liu D, Tessier A, Hutchison MJ, Murray D (2000). ERCC1 expression as a molecular marker of cisplatin resistance in human cervical tumor cells. Int J Cancer.

[10] Shirota Y, Stoehlmacher J, Brabender J, Xiong YP, Uetake H, Danenberg KD (2001). ERCC1 and thymidylate synthase mRNA levels predict survival for colorectal cancer patients receiving combination oxaliplatin and fluorouracilchemotherapy. J Clin Oncol.

[11] Lord RVN, Brabender J, Gandara D, Alberola V, Camps C, Domine M (2002). Low *ERCC1* expression correlates with prolonged survival after cisplatin plus gemcitabine chemotherapy in non-small cell lung cancer. Clin Cancer Res.

[12] Shen MR, Jones IM, Mohrenweiser H (1998). Nonconservative amino acid substitution variants exist at polymorphic frequency in DNA repair genes in healthy humans. Cancer Res.

[13] Se’ve P, Dumontet C (2008). Is class III A-tubulin a predictive factor in patients receiving tubulin-binding agents?. Lancet Oncol.

[14] Fung KL, Gottesman MM (2009). A synonymous polymorphism in a common MDR1 (ABCB1) haplotype shapes protein function. Biochim Biophys Acta.

[15] Kimchi-Sarfaty C, Oh JM, Kim IW, Sauna ZE, Calcagno AM, Ambudkar SV (2007). A “silent” polymorphism in the MDR1 gene changes substrate speciWcity. Science.

[16] Zhou J. Multi-drug resistance in cancer. 1st ed. New York : Humana Press;2010.

[17] Ferrandina G, Lauriola L, Distefano MG, Zannoni GF, Gessi M, Legge F (2002). Increased cyclooxygenase-2 expression is associated with chemotherapy resistance and poor survival in cervical cancer patients. J Clin Oncol.

[18] Modugno F, Ness RB, Chen C, Weiss NS (2005). Inflammation and endometrial cancer: a hypothesis. Cancer Epidemiol Biomark Prev.

[19] Goswami B, Rajappa M, Sharma M, Sharma A (2008). Inflammation: its role and interplay in the development of cancer, with special focus on gynecological malignancies. Intern J Gynecol Cancer.

[20] Dudley ME, Wunderlich JR, Robbins PF, Yang JC, Hwu P, Schwartzentruber DJ (2002). Cancer regression and autoimmunity in patients after clonal repopulation with antitumor lymphocytes. Science.

[21] Sato E, Olson SH, Ahn J, Bundy B, Nishikawa H, Qian F (2005). Intraepithelial CD81 tumor infiltrating lymphocytes and a high CD81/regulatory T cell ratio are associated with favorable prognosis in ovarian cancer. Proc Natl Acad Sci U S A.

[22] Prall F, Duhrkop T, Weirich V, Ostwald C, Lenz P, NizzeHet al. Prognostic role of CD81 tumor-infiltrating lymphocytes in stage III colorectal cancer with and without microsatellite instability. Hum Pathol 2004; 35: 808–816. 10.1016/j.humpath.2004.01.022 15257543

[23] Fukunaga A, Miyamoto M, Cho Y, Murakami S, Kawarada Y, Oshikiri T (2004). CD81 tumor-infiltrating lymphocytes together with CD41 tumor-infiltrating lymphocytes and dendritic cells improve the prognosis of patients with pancreatic adenocarcinoma. Pancreas.

[24] Shah W, Yan X, Jing L, Zhou Y, Chen H, Wang Y (2011). A reversed CD4/CD8 ratio of tumor-infiltrating lymphocytes and a high percentage of CD41FOXP31 regulatory T cells are significantly associated with clinical outcome in squamous cell carcinoma of the cervix. Cellular & Molecular Immunology.

[25] Mountzios G, Dimopoulos MA, Bamias A, Vourli G, Kalofonos H, Aravantinos G (2009). Randomized multicenter phase II trial of cisplatin and ifosfamide with or without paclitaxel in recurrent or metastatic carcinoma of the uterine cervix: a Hellenic Cooperative Oncology Group (HeCOG) study. Ann Oncol.

[26] Jaffe CC (2006). Measures of response: RECIST, WHO, and new alternatives. J Clin Oncol.

[27] Kalikaki A, Kanaki M, Vassalou H, Souglakos J, Voutsina A, Georgoulias V (2009). DNA repair gene polymorphisms predict favorable clinical outcome in advanced non-small-cell lung cancer. Clin Lung Cancer.

[28] Yin Z (2009). ERCC2, ERCC1 polymorphisms and haplotypes, cooking oil fume and lung adenocarcinoma risk in Chinese non-smoking females. J Exp Clin Cancer Res.

[29] Tanabe M, Ieiri I, Nagata N, Inoue K, Ito S, Kanamori Y (2001). Expression of P-glycoprotein in human placenta: relation to genetic polymorphism of the multidrug resistance (MDR)-1 gene. J Pharmacol Exp Ther.

[30] Cascorbi I, Gerloff T, Johne A, Meisel C, Hoffmeyer S, Schwab M (2001). Frequency of single nucleotide polymorphisms in the P-glycoprotein drug transporter MDR1 gene in white subjects. Clin Pharmacol Ther.

[31] Britten RA, Liu D, Tessier A, Hutchison MJ, Murray D (2000). ERCC1 expression as a molecular marker of cisplatin resistance in human cervical tumor cells. Int J Cancer.

[32] Zwenger AO, Grosman G, Iturbe J, Leone J, Vallejo CT, Leone JP (2015). Expression of ERCC1 and TUBB3 in locally advanced cervical squamous cell cancer and its correlation with different therapeutic regimens. Int J Biol Markers.

[33] Ferrandina G, Martinelli E, Zannoni GF, Distefano M, Paglia A, Ferlini C (2007). Expression of class III beta tubulin in cervical cancer patients administered preoperative radiochemotherapy: correlation with response to treatment and clinical outcome. Gynecol Oncol.

[34] Doll CM, Aquino-Parsons C, Pintilie M, Klimowicz AC, Petrillo SK, Milosevic M (2013). The significance of tumoral ERCC1 status in patients with locally advanced cervical cancer treated with chemoradiation therapy: a multicenter clinicopathologic analysis. Int J Radiat Oncol Biol Phys.

[35] Doll CM, Prystajecky M, Eliasziw M, Klimowicz AC, Petrillo SK, Craighead PS (2010). Low ERCC1 mRNA and protein expression are associated with worse survival in cervical cancer patients treated with radiation alone. Radiother Oncol.

[36] Hasegawa K, Kato R, Torii Y, Ichikawa R, Oe S, Udagawa Y (2011). The relationship between ERCC1 expression and clinical outcome in patients with FIGO stage I to stage II uterine cervical adenocarcinoma. Int J Gynecol Cancer.

[37] Park JS, Jeon EK, Chun SH, Won HS, Lee A, Hur SY (2011). Hong SH ERCC1 (excision repair cross-complementation group 1) expression as a predictor for response of neoadjuvant chemotherapy for FIGO stage 2B uterine cervix cancer. Gynecol Oncol.

[38] Muallem MZ, Marnitz S, Richter R, Köhler C, Sehouli J (2014). Arsenic R ERCC1 expression as a predictive marker of cervical cancer treated with cisplatin-based chemoradiation. Anticancer Res.

[39] Lee SM, Falzon M, Blackhall F (2017). Randomized prospective biomarker trial of ERCC1 for comparing platinum and nonplatinum therapy in advanced non-small-cell lung cancer: ERCC1 trial (ET). J Clin Oncol.

